# Breast cancer screening in women aged 75 and older: Insights from an aging region in Japan

**DOI:** 10.1016/j.pmedr.2025.103273

**Published:** 2025-10-09

**Authors:** Kaolu Sato, Akihiko Furuta, Haruhiko Shindo, Mii Shibahara, Sakurako Ishikawa, Miku Kurusu, Minoru Miyashita

**Affiliations:** a Department of Breast Surgery, Japan Red Cross Ishinomaki Hospital, Japan; b Department of Breast and Endocrine Surgical Oncology, Tohoku University School of Medicine, Japan

**Keywords:** Breast cancer screening, Elderly women, Japan, Overdiagnosis, Mortality, Public health, Population aging

## Abstract

**Objective:**

In Japan, breast cancer incidence and mortality are rising with population aging. Screening may be associated with lower mortality but raises concern about overdiagnosis in older adults. Current guidelines provide no clear upper age limit. This study assessed screening in women aged ≥75 in an aging region.

**Methods:**

We retrospectively analyzed 289 women aged 75–98 years (median 81) diagnosed with breast cancer at Japan Red Cross Ishinomaki Hospital (2011−2020). Patients were classified as screened (population-based screening) or non-screened (symptom- or incidentally-detected). The primary outcome was all-cause mortality. Comparisons used Mann–Whitney U, chi-square, or Fisher's exact tests. Survival was analyzed by Kaplan–Meier and log-rank tests; prognostic factors by Cox models.

**Results:**

Of 289 patients, 46 (15.9 %) were screened and 243 (84.1 %) non-screened. Screened patients were younger, had smaller tumors, fewer lymph node metastases, and more surgery. Screening was associated with lower mortality in univariate but not multivariable analysis. No breast cancer-specific deaths occurred in the screened group versus 25 (10.3 %) in the non-screened group (*p* = 0.02).

**Conclusions:**

Screening was not an independent predictor of survival but was associated with absence of breast cancer-specific deaths, supporting further studies in elderly populations.

## Highlights

• Analyzed breast cancer screening in Japan's aging region.
• No breast cancer-specific deaths in the screened group.
• All-cause mortality was lower in the screened group, with age differences noted.

## 1 Objective

In Japan, the incidence and mortality rates of breast cancer have been rising (International Agency for Research on Cancer (IARC), 2025), paralleling the rapid aging of the population, which has become a significant social concern, and our hospital is located in one of the regions in Japan with the highest proportion of elderly residents (*Ishinomaki city*, 2025). The purpose of screening is to detect breast cancer at an earlier stage, and which is expected to contribute to reductions in breast cancer mortality (Freedman et al., 2017; Trentham-Dietz et al., 2016; Trentham-Dietz et al., 2024). Among older adults, however, overdiagnosis—the detection of cancers that would not have caused harm during the patient's lifetime—is a significant concern (Richman et al., 2023; Lee et al., 2012; Zahl, 2018; Etzioni et al., 2013). Reports suggest that over half of women aged 85 and older who undergo screening may be overdiagnosed (Richman et al., 2023; Braithwaite et al., 2016). Overdiagnosis can lead to unnecessary procedures, physical and psychological distress, and financial burden (Etzioni et al., 2013; Pickles et al., 2022; Gøtzsche and Jørgensen, 2013; Molina et al., 2017).

Based on such concerns, the United States Preventive Services Task Force has concluded that there is insufficient evidence to recommend routine breast cancer screening in women over 75 (Freedman et al., 2017; Richman et al., 2023). However, other guidelines suggest continued biennial screening up to age 80 in healthy individuals, defined as those with a life expectancy of over 10 years (Freedman et al., 2017; Richman et al., 2023; Lee et al., 2012; Schousboe et al., 2022; Schoenborn et al., 2017; Mack et al., 2018). In Japan, the 2018 edition of the Japanese Breast Cancer Society Clinical Practice Guidelines stated that the optimal upper age limit for breast cancer screening was approximately 75 years. However, this recommendation was removed in the current 2022 edition (Japanese Breast Cancer Society Clinical Practice Guidelines, 2025).

In addition to the lack of clear guidance in the current guidelines, clinical experience with treating elderly breast cancer patients reveals that the complexity of their individual circumstances and surrounding environment further complicates the issue of screening in this population. From a clinical perspective, elderly patients are highly diverse in terms of health status and values, making it difficult to predict life expectancy accurately. Therefore, shared and personalized decision-making is essential for this population (Smith et al., 2021; Schoenborn et al., 2019; Jayasekera et al., 2024).

This study aimed to explore the role of breast cancer screening in women aged 75 and older in a highly aged region of Japan from a clinical perspective, in an effort to bridge the gap between public health recommendations and real-world clinical practice.

## 2 Methods

We retrospectively analyzed medical records of women aged 75 or older who were newly diagnosed with breast cancer and received primary treatment or treatment planning at Department of Breast Surgery, Japan Red Cross Ishinomaki Hospital between September 2011 and December 2020. Data were anonymized and collected from electronic health records. For patients who had not visited our hospital for more than five years, follow-up information was obtained via telephone survey.

Patients were classified into two: *screened* (diagnosed through biennial nationwide population-based breast cancer screening targeting all women aged 40 years and older) and *non-screened* (diagnosed due to symptoms or incidental findings on imaging for other diseases such as Computed Tomography). Interval cancers (defined as cancers detected within two years after the most recent screening) were classified into either the symptomatic group or the incidentally detected group.

The primary outcome was all-cause mortality. Stage was classified according to the eighth edition of the Union for International Cancer Control classification. Comparisons between the *screening* and *non-screening* groups were performed using the Mann–Whitney *U* test for age at initial visit, follow-up duration, and invasive tumor size, and the chi-square test or Fisher's exact test for other variables.

Survival analysis was conducted using the Kaplan-Meier method, with log-rank tests to compare groups. Multivariable analysis using the Cox proportional hazards model included invasive tumor size, lymph node involvement, distant metastasis, and treatment variables.

All statistical analyses were performed using R (R Core Team, 2025), with a significance level set at *p* < 0.05. The data supporting the findings of this study are openly available in the Mendeley Data repository (Sato, 2025) at https://doi.org/10.17632/rwgks8m5w9.2. The study was approved by the hospital ethics committee (Approval No. 25–09).

## 3 Results

Clinicopathological characteristics are summarized in Table 1. A total of 289 patients met the inclusion criteria. Among them, 46 (15.9 %) were in the screened group and 243 (84.1 %) in the non-screened group. Patient ages ranged from 75 to 98 years, with a median age of 81 years. The screened group was significantly younger than the non-screened group (*p* < 0.01). In the non-screening group, 217 patients (89.3 %) presented with symptoms such as awareness of a breast lump, while 26 patients (10.7 %) were referred after abnormalities were incidentally detected on imaging studies performed for other diseases, including Computed Tomography. Cases with comorbidities were common in both groups: 41 cases (89.1 %) in the screened group and 211 cases (86.8 %) in the non-screened group, with no significant difference observed (*p* = 0.62).

**Table 1:** Clinicopathological Characteristics, Treatments, and Outcomes of Screened and Non-Screened Patients in Japanese women 75 years and older (2011–2020).

		Total	Screened	Non-screened	*p*-value	
Number of cases		289	46	243		
Age at initial visit	Minimum	75	75	75	<0.01	**
	Maximum	98	90	98		
	Median	81	78	82		
Method of Detection	Screening	46	46†	0	<0.01	***
	Symptomatic	217	0	217		
	Incidental	26	0	26		
Months of follow-up	Minimum	0	10	0	0.02	**
	Maximum	159	150	159		
	Median	62	68.5	59		
	Unknown	1	0	1		
Comorbidities	None	8	2	6	0.62	***
	Yes	252	41	211		
	Unknown	29	3	26		
Invasive tumor size (cm)	Minimum	0	0	0	<0.01	**
	Maximum	11	4.7	11		
	Median	1.8	0.8	2.1		
	Unknown	14	0	14		
Lymph node metastasis	Negative	195	40	155	0.03	*
	Positive	65	5	60		
	Unknown	29	1	28		
Distant metastasis	Negative	239	43	196	0.594	***
	Positive	6	0	6		
	Unknown	44	3	41		
Stage	0	17	6	11	<0.01	***
	I	107	31	76		
	II	86	5	81		
	III	15	0	15		
	IV	6	0	6		
	Unknown	58	4	54		
Subtype	HR+/HER-	210	38	172	0.43	***
	HR+/HER+	15	2	13		
	HR-/HER+	12	0	12		
	HR-/HER-	34	5	29		
	Unknown	18	1	17		
Bilateral	Yes	12	1	11	0.70	***
	No	277	45	232		
Surgical intervention	Yes	210	43	167	<0.01	*
	No	79	3	76		
Hormone therapy	Yes	180	34	146	0.11	*
	No	109	12	97		
Chemotherapy	Yes	13	0	13	0.23	***
	No	276	46	230		
Breast cancer-specific death		25	0	25	0.02	***
All-cause mortality		71	2	69	<0.01	*

*Chi-squared test, **Mann–Whitney U test, *** Fisher's exact test.

This table summarizes the baseline characteristics, tumor biology, treatment patterns, and clinical outcomes of patients aged 75 years and older with breast cancer, stratified by screening history. Significant differences were observed between groups in invasive tumor size, lymph node and distant metastasis status, surgical intervention, and mortality outcomes. No significant difference was found in subtype distribution, hormone therapy or chemotherapy administration.

†Includes one patient who had symptoms but was diagnosed after screening.

*HR*: Hormone Receptor expression, *HER*: Human epidermal growth factor receptor 2 expression.

The median invasive tumor size was significantly smaller in the screened group (0.8 cm) than in the non-screened group (2.1 cm, *p* < 0.01). Lymph node metastases were present in 10.9 % of the screened group and 24.7 % of the non-screened group (*p* = 0.03). No distant metastases were observed in the screened group, whereas six cases (2.5 %) were found in the non-screened group.

When considered by stage, Stage I was the most frequent in the screened group, observed in 31 patients (67.4 %), and no cases of Stage III or higher were identified (0 %). In contrast, in the non-screened group, Stage II was the most common, found in 81 patients (33.3 %), and 21 patients (8.6 %) were diagnosed with Stage III or higher disease.

Regarding subtypes, the distribution was as follows: in the screened group, hormone receptor-positive/HER2-negative (HR+/HER2−) in 38 cases (82.6 %), HR+/HER2+ in five cases (10.9 %), HR−/HER2+ in zero cases (0 %), and HR−/HER2− in two cases (4.3 %); in the non-screened group, HR+/HER2− in 172 cases (70.8 %), HR+/HER2+ in 13 cases (5.3 %), HR−/HER2+ in 12 cases (4.9 %), and HR−/HER2− in 29 cases (11.9 %). There was no significant difference in subtype distribution between the groups (*p* = 0.43).

Regarding treatment, surgical intervention was performed in 43 patients (93.5 %) in the screened group and in 167 patients (68.7 %) in the non-screened group, showing a significantly higher rate in the screened group (*p* < 0.01). Hormone therapy was administered in 34 patients (73.9 %) in the screened group and in 146 patients (60.1 %) in the non-screened group, with no significant difference (*p* = 0.11). Chemotherapy was administered in none of the screened patients (0 %) and in 13 patients (5.3 %) in the non-screened group; both rates were low.

All-cause mortality occurred in two cases (4.3 %) in the screened group and 69 cases (28.4 %) in the non-screened group, showing a significant difference (*p* < 0.01). Notably, there were no breast cancer-specific deaths in the screened group (0 %), whereas 25 cases (10.3 %) were observed in the non-screened group (*p* = 0.02).

Supplementary Table 1 shows the reclassification of the screened and non-screened groups according to the method of detection (screen-detected, symptomatic, or incidentally detected). One patient who was aware of symptoms but presented to our hospital through screening was excluded, leaving 288 cases for analysis. Comparison among the three groups revealed significant differences in age at initial visit (*p* < 0.01), invasive tumor size (p < 0.01), stage (p < 0.01), surgical intervention (p < 0.01), breast cancer-specific death (*p* = 0.03), and all-cause mortality (p < 0.01). The incidentally detected group showed a higher age at initial visit, similar to the symptomatic group, but in terms of invasive tumor size, disease stage, surgical intervention, breast cancer-specific death, and all-cause mortality, their clinicopathological characteristics were comparable to those of the screen-detected group. Interval cancers were identified in 16 cases (7.4 %) of the symptomatic group and two cases (7.7 %) of the incidentally detected group. However, recent screening information was unavailable for 44 cases (20.3 %) in the symptomatic group and four cases (15.4 %) in the incidentally detected group.

**Fig. 1:**
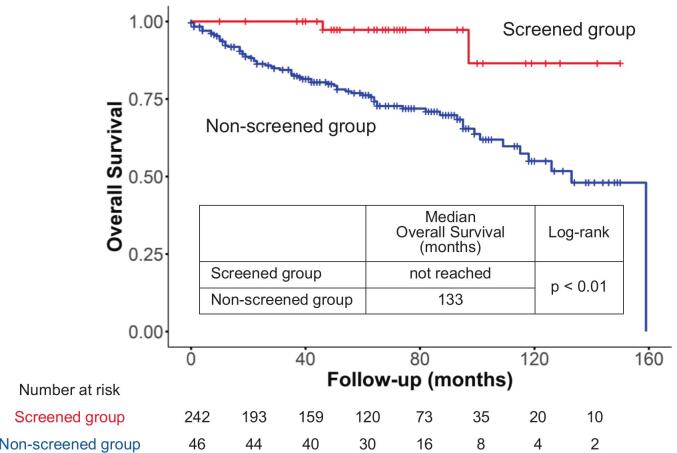
Kaplan–Meier Survival Curves for breast cancer detection, Comparing Screened and Non-Screened Groups in Japanese women 75 years and older (2011–2020). Kaplan–Meier survival analysis demonstrates a significantly longer overall survival in the screened group compared to the non-screened group (median overall survival not reached within the follow-up period vs. 133 months, respectively; log-rank *p* < 0.01).

Kaplan-Meier analysis (Fig. 1) showed a median overall survival of 133 months in the non-screened group, whereas in the screened group the median was not reached within the follow-up period (*p* < 0.01).

**Fig. 2:**
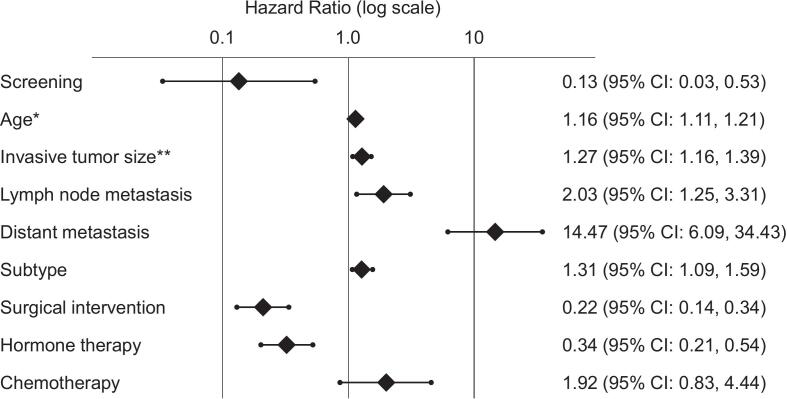
Forest Plot of Univariate Cox Proportional Hazards Analysis for All-Cause Mortality in Japanese women 75 years and older (2011–2020). Univariate Cox regression analysis revealed that breast cancer screening was significantly associated with a reduced risk of all-cause mortality. * HR per one-year increase in age. ** HR per one-cm increase in size.

In univariate analysis (Fig. 2), screening was significantly associated with a lower risk of all-cause mortality (HR 0.13, 95 % CI: 0.03, 0.53). Other significant factors for all-cause mortality included age (per one-year increase: HR 1.16, 95 % CI: 1.11, 1.21), invasive tumor size (per one-cm increase: HR 1.27, 95 % CI: 1.06, 1.39), lymph node metastasis (HR 2.03, 95 % CI: 1.25, 3.31), distant metastasis (HR 14.47, 95 % CI: 6.09, 34.43), subtype (HR 1.31, 95 % CI: 1.09, 1.59), surgical intervention (HR 0.22, 95 % CI: 0.14, 0.35), and hormone therapy (HR 0.34, 95 % CI: 0.21, 0.54).

**Fig. 3:**
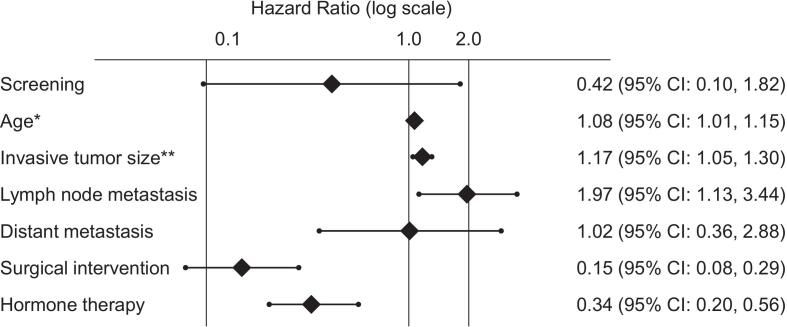
Forest Plot of Multivariable Cox Proportional Hazards Analysis for All-Cause Mortality in Japanese women 75 years and older (2011–2020). In multivariable Cox regression analysis, breast cancer screening demonstrated a trend toward reduced mortality, this did not reach statistical significance. * HR per one-year increase in age. ** HR per one-cm increase in size.

In multivariable analysis (Fig. 3), age (per one-year increase: HR 1.08, 95 % CI: 1.01, 1.15), invasive tumor size (per one-cm increase: HR 1.17, 95 % CI: 1.05, 1.30), lymph node metastasis (HR 1.97, 95 % CI: 1.13, 3.44), surgical intervention (HR 0.15, 95 % CI: 0.08, 0.29), and hormone therapy (HR 0.34, 95 % CI: 0.20, 0.56) remained statistically significant prognostic factors for all-cause mortality. Screening showed a trend toward lower all-cause mortality (HR 0.42) but did not reach statistical significance (95 % CI: 0.10, 1.82).

## 4 Discussion

Regarding the treatment section, the rate of hormone therapy was high in both the screened (73.9 %) and non-screened (60.1 %) groups. Previous studies have also reported that the use of hormone therapy alone increases significantly with age (İnal et al., 2014).

This is likely attributable to the biological characteristics of the cancers, in which hormone receptor-positive/HER2-negative (HR+/HER2−) was the most prevalent subtype. This finding aligns with previous studies reporting that breast cancers in elderly patients tend to be more likely hormone receptor-positive and less likely to express HER2 compared to those in younger individuals (İnal et al., 2014; Gennari et al., 2004).

In contrast, chemotherapy was administered at a very low rate in both groups (0 % in the screened group and 5.3 % in the non-screened group). This result reflects the clinical tendency to avoid chemotherapy in elderly patients due to concerns over comorbidities and potential adverse events (Gennari et al., 2004; Varghese and Wong, 2018; Blackmore et al., 2018; Schonberg et al., 2010). Some studies have identified age as the strongest determinant for omitting chemotherapy (Blackmore et al., 2018). However, there are reports suggesting that chemotherapy may still be beneficial for elderly patients with biologically aggressive tumors (Varghese and Wong, 2018), highlighting the complexity of treatment decisions in this population.

A comparison of the screened and non-screened groups revealed that the screened group was significantly younger (median age: 78 vs. 82 years, *p* < 0.01), had significantly smaller tumors (median 0.8 cm vs. 2.1 cm, p < 0.01), and a lower incidence of lymph node metastases (10.9 % vs. 24.7 %, *p* = 0.03). Previous reports have noted that elderly patients—particularly those beyond routine screening age—are more likely to be diagnosed at a more advanced stage, with larger tumors and greater nodal involvement (Welch et al., 2016). This study similarly found that patients aged 80 or older were more likely to be diagnosed based on symptoms rather than screening, which may explain the observed differences in tumor size.

Our screened and non-screened groups were reclassified according to the categories used in previous studies: “screen-detected,” “symptomatic,” and “incidentally detected.” (Ghate et al., 2024; Munir et al., 2025) The screened group corresponded closely to the “screen-detected” category. Consistent with previous reports, “screen-detected” cases in our study tended to present at lower stages, whereas “symptomatic” cases were more often diagnosed at higher stages. “Incidentally detected” cases showed characteristics similar to those of the “screen-detected” group, but as they accounted for fewer than 10 % of all cases, their interpretation requires caution. We also identified interval cancers, although their interpretation is limited by the lack of recent screening information in a subset of patients.

With respect to treatment, surgical intervention was significantly more frequent in the screened group (93.5 % vs. 68.7 %, *p* < 0.01). This may reflect both patient-related factors—such as individuals in better health being more likely to undergo screening (Schoenborn et al., 2017; Smith et al., 2021)—and clinician hesitancy to recommend surgery for elderly patients with multiple comorbidities, mirroring patterns observed in the underutilization of chemotherapy (Varghese and Wong, 2018; Schonberg et al., 2010). Even after adjusting for comorbidities and cancer stage, studies have shown that the likelihood of undergoing surgery decreases significantly with age (Blackmore et al., 2018). Our results also showed no difference in comorbidity rates between the screened and non-screened groups (89.1 % vs. 86.8 %, *p* = 0.62).

In univariate analysis, the screened group was observed to have significantly longer survival, with a notably lower hazard ratio (HR 0.13, *p* < 0.01). However, in multivariable analysis including age, invasive tumor size, lymph node and distant metastases, hormone therapy, and surgical intervention, the association between screening and all-cause mortality was no longer statistically significant, though a favorable trend was observed (HR 0.42, *p* = 0.25). Age, invasive tumor size, lymph node metastasis, and surgical intervention remained significant predictors of mortality. This observation suggests that elderly individuals may be self-selecting out of breast cancer screening, possibly due to personal concerns or perceived barriers.

Potential harms of cancer screening must be considered. The most significant is overdiagnosis—the detection and treatment of cancers that would not have caused clinical symptoms or death during the patient's lifetime. Such cases can lead to unnecessary physical, psychological, and financial burdens, particularly for elderly patients (Freedman et al., 2017; Richman et al., 2023; Lee et al., 2012; Etzioni et al., 2013; Smith et al., 2021; Walter et al., 2005; Moss et al., 2020). Therefore, the effectiveness of cancer screening in this population should be primarily evaluated based on all-cause mortality.

Nonetheless, breast cancer has disease-specific characteristics that warrant careful attention. In terminal-stage disease, progressive local tumors can cause malignant skin ulceration, which can severely affect quality of life (Abdallah et al., 2023; Maida et al., 2009; Tan, 2016). In this study, there were no breast cancer-specific deaths in the screened group (0 %), whereas 25 deaths (10.3 %) occurred in the non-screened group (*p* = 0.02). As Welch and others have suggested, the reduction in breast cancer mortality may be more attributable to advances in treatment rather than early detection via screening (Welch et al., 2016). Indeed, other studies have found no statistically significant reduction in breast cancer-specific mortality from screening in older women (Richman et al., 2023; Walter et al., 2005).

However, the absence of breast cancer-specific deaths in our study, combined with the limited treatment options often available for elderly patients, suggests this finding warrants careful consideration. From the perspective of avoiding malignant skin ulceration in end-of-life care, our findings suggest that screening may still provide meaningful benefits for elderly patients. Moreover, beyond the end-of-life setting, maintaining dignity throughout aging is of paramount importance. In discussions on the optimal age for screening, it is essential to balance survival benefits with quality of life and respect for patient autonomy.

Naturally, our study has several limitations. As a retrospective observational study from a single institution, selection bias and unmeasured confounding factors cannot be ruled out. Therefore, to further elucidate the significance of breast cancer screening in the elderly population, large-scale studies utilizing data from the National Clinical Database or prospective trials are warranted. Such efforts may help address the various public health challenges associated with breast cancer screening in older adults.

## 5 Conclusion

This study showed that breast cancer screening was associated with a lower risk of all-cause mortality in elderly women in univariate analysis, though this association weakened in multivariable analysis. Importantly, no breast cancer-specific deaths were observed in the screened group, suggesting that screening may still play a beneficial role in selected individuals.

The value of breast cancer screening in older adults remains a matter of ongoing debate, particularly in relation to potential harms such as overdiagnosis. We hope that our data, derived from a region in Japan facing significant population aging, will serve as a valuable contribution toward addressing the complex issues surrounding breast cancer screening in the elderly.

## Declaration of generative AI in scientific writing

During the preparation of this work the author used ChatGPT (OpenAI) and NotebookLM (Google) in order to improve the readability and language of the manuscript. After using these tools, the author reviewed and edited the content as needed and takes full responsibility for the content of the published article.

## CRediT authorship contribution statement

**Kaolu Sato:** Writing – original draft, Visualization, Methodology, Investigation, Formal analysis, Data curation, Conceptualization. **Akihiko Furuta:** Writing – review & editing, Supervision, Investigation, Conceptualization. **Haruhiko Shindo:** Writing – review & editing, Investigation, Conceptualization. **Mii Shibahara:** Writing – review & editing, Investigation, Conceptualization. **Sakurako Ishikawa:** Writing – review & editing, Investigation, Conceptualization. **Miku Kurusu:** Writing – review & editing, Investigation, Conceptualization. **Minoru Miyashita:** Writing – review & editing, Validation, Investigation, Formal analysis, Conceptualization.

## Funding information

The authors received no financial support for the research, author ship, and/or publication of this article.

## Declaration of competing interest

The authors declare that they have no known competing financial interests or personal relationships that could have appeared to influence the work reported in this paper.

## Data Availability

The data supporting the findings of this study are openly available in the Mendeley Data repository at Doi: 10.17632/rwgks8m5w9.2

## Appendix A Supplementary data

Supplementary material 1: Supplementary Table S1. Clinicopathological characteristics, treatments, and outcomes by method of detection (screened, symptomatic, and incidentally detected) in Japanese women aged 75 years and older (2011–2020). Image 1

Supplementary material 2: The table presents data comparing clinical and pathological variables, treatment modalities, and outcomes across the three detection groups. Image 2
