# Supplementary material for: Breast cancer screening in women aged 75 and older: Insights from an aging region in Japan
Source: Prev Med Rep. 2025 Oct 9;59:103273. doi: 10.1016/j.pmedr.2025.103273 (PMC12550720; doi:10.1016/j.pmedr.2025.103273)
Supplement: Supplementary file 1 — Supplementary material 1: Supplementary Table S1. Clinicopathological characteristics, treatments, and outcomes by method of detection (screened, symptomatic, and incidentally detected) in Japanese women aged 75 years and older (2011–2020). [file mmc1.docx]

**Suppl. Table 1. Clinicopathological Characteristics, Treatments, and Outcomes by Method of Detection: Screened, Symptomatic, and Incidentally Detected Groups in Japanese women 75 years and older (2011-2020)**

|  | |  | Total | Screened | Non-screened | | p-value | |
| --- | --- | --- | --- | --- | --- | --- | --- | --- |
|  | |  |  |  | Symptomatic | Incidental |  |  |
| Number of cases | |  | 288 | 45 | 217 | 26 |  |  |
| Age at initial visit | | Minimum | 75 | 75 | 75 | 76 | <0.01 | ** |
|  | | Maximum | 98 | 90 | 95 | 98 |  |  |
|  | | Median | 81 | 78 | 82 | 81.5 |  |  |
| Months of follow-up | | Minimum | 0 | 10 | 0 | 4 | 0.05 | ** |
|  | | Maximum | 159 | 150 | 159 | 127 |  |  |
|  | | Median | 62 | 68 | 60.5 | 44 |  |  |
|  | | Unknown | 1 | 0 | 1 | 0 |  |  |
| Interval cancer† | | YES |  |  | 16 | 2 |  |  |
|  | | No |  |  | 157 | 20 |  |  |
|  | | Unknown |  |  | 44 | 4 |  |  |
| Comorbidities | | None | 8 | 2 | 5 | 1 | 0.26 | * |
|  | | Yes | 251 | 40 | 186 | 25 |  |  |
|  | | Unknown | 29 | 3 | 26 | 0 |  |  |
| Invasive tumor size (cm) | | Minimum | 0 | 0 | 0 | 0 | <0.01 | ** |
|  | | Maximum | 11 | 4.7 | 11 | 5 |  |  |
|  | | Median | 1.8 | 0.9 | 2.1 | 1.4 |  |  |
|  | | Unknown | 14 | 0 | 14 | 0 |  |  |
| Lymph node metastasis | | Negative | 194 | 39 | 139 | 16 | 0.06 | * |
|  | | Positive | 65 | 5 | 52 | 8 |  |  |
|  | | Unknown | 29 | 1 | 26 | 2 |  |  |
| Distant metastasis | | Negative | 238 | 42 | 173 | 23 | 0.32 | * |
|  | | Positive | 6 | 0 | 6 | 0 |  |  |
|  | | Unknown | 44 | 3 | 38 | 3 |  |  |
| Stage | | 0 | 17 | 6 | 9 | 2 | <0.01 | * |
|  | | I | 106 | 30 | 64 | 12 |  |  |
|  | | II | 86 | 5 | 75 | 6 |  |  |
|  | | III | 15 | 0 | 14 | 1 |  |  |
|  | | IV | 6 | 0 | 6 | 0 |  |  |
|  | | Unknown | 58 | 4 | 49 | 5 |  |  |
| Subtype | | HR+/HER- | 209 | 37 | 151 | 21 | 0.42 | * |
|  | | HR+/HER+ | 15 | 2 | 13 | 0 |  |  |
|  | | HR-/HER+ | 12 | 0 | 12 | 0 |  |  |
|  | | HR-/HER- | 34 | 5 | 26 | 3 |  |  |
|  | | Unknown | 18 | 1 | 15 | 2 |  |  |
| Bilateral | | Yes | 12 | 1 | 10 | 1 | 0.88 | * |
|  | | No | 276 | 44 | 207 | 25 |  |  |
| Surgical intervention | | Yes | 209 | 42 | 148 | 19 | <0.01 | * |
|  | | No | 79 | 3 | 69 | 7 |  |  |
| Hormone therapy | | Yes | 180 | 33 | 130 | 16 | 0.24 | * |
|  | | No | 109 | 12 | 87 | 10 |  |  |
| Chemotherapy | | Yes | 13 | 0 | 13 | 0 | 0.10 | * |
|  | | No | 276 | 45 | 204 | 26 |  |  |
| Breast cancer-specific death | | | 25 | 0 | 24 | 1 | 0.03 | * |
| All-cause mortality | | | 70 | 1 | 63 | 6 | <0.01 | * |
|  | ＊Chi-squared test, **Kruskal-Wallis test | | | | | | | |

This table shows the clinicopathological characteristics according to the method of detection. The detection methods were categorized into three groups:

・Screening group: Patients diagnosed after participating in community-based breast cancer screening.

・Symptomatic group: Patients diagnosed after presenting with clinical symptoms.

・Incidental-detected group: Patients diagnosed incidentally when abnormalities were found during imaging for other diseases (e.g., Computed Tomography).

In this study, cases detected through screening tended to be diagnosed at earlier stages, whereas symptomatic cases tended to present with more advanced disease. Incidental-detected cases showed characteristics similar to those of the screening group; however, as they accounted for fewer than 10% of all cases, these findings should be interpreted with caution.

†Interval cancer was defined as cancer detected within two years after the most recent screening.

*HR*: Hormone Receptor expression, *HER*: Human epidermal growth factor receptor 2 expression.
