# Supplementary material for: Breast cancer screening in women aged 75 and older: Insights from an aging region in Japan
Source: Prev Med Rep. 2025 Oct 9;59:103273. doi: 10.1016/j.pmedr.2025.103273 (PMC12550720; doi:10.1016/j.pmedr.2025.103273)
Supplement: Supplementary file 2 — Supplementary material 2: The table presents data comparing clinical and pathological variables, treatment modalities, and outcomes across the three detection groups. [file mmc2.docx]

List of Revisions

We have made the following revisions to the manuscript:

Highlights：

- #3: To maintain neutrality consistent with the observational nature of the study and to make the sentence self-contained, we revised “All-cause mortality was lower in screened group, but age difference influenced.” to “All-cause mortality was lower in the screened group, with age differences noted.”

This revision also complies with the journal’s guideline limiting each highlight to 85 characters including spaces.

Manuscript：

Abstract

- Page 1, lines 13–14: Revised “associate” to “was associated.”

Results

- Page 4, line 11: Revised “screening significantly associated …” to “screening was significantly associated ….”

Figure Legends

- Figure 1: As instructed, we added “for breast cancer detection” to the title and revised it from “Kaplan–Meier Survival Curves Comparing Screened and ….” to “Kaplan–Meier Survival Curves for breast cancer detection, Comparing Screened and ….”
